# Association of atrial cardiopathy and residual shunt after patent foramen ovale closure in patients experiencing migraine

**DOI:** 10.1186/s40001-025-02423-8

**Published:** 2025-03-14

**Authors:** Xin Gao, Xinxin Zhang, Wei Song, Yan Liu, Ran Guo, Yinong Jiang

**Affiliations:** Department of Cardiology, Institute of Cardiovascular Diseases, The First Affiliated Hospital of Dalian Medical University, 193 United Road, Dalian, 116021 Liaoning China

**Keywords:** Atrial cardiopathy, Patent foramen ovale, Residual shunt, Migraine relief, Outcomes of PFO closure

## Abstract

**Aims:**

Migraine is a prevalent and incapacitating condition. The association between patent foramen ovale (PFO) and migraine-type headaches has been extensively documented. In clinical practice, patients may observe residual shunting after PFO closure. Nevertheless, the underlying mechanisms affecting residual shunting after PFO closure remain unclear. Recent studies have identified left atrial abnormalities, specifically atrial cardiopathy, as an independent risk factor for the development of atrial fibrillation (AF), left atrial thrombosis, and subsequent stroke. To that end, the present study aims to investigate the relationship between residual shunt occurrence after PFO closure and atrial cardiopathy.

**Methods:**

A retrospective analysis comparing postoperative residual shunts in patients with and without atrial cardiopathy was conducted. The study cohort consisted of 174 patients with severe migraine and confirmed right-to-left shunt (RLS) (grades II-IV) who voluntarily opted for PFO closure between April 1, 2021, and December 31, 2022. Enrolled patients were categorized into two groups: PFO with or without atrial cardiopathy.

**Results:**

A total of 174 migraineurs who underwent PFO closure (PFO with atrial cardiopathy group, *n =* 20; PFO without atrial cardiopathy group, *n =* 154) were included. Compared to patients without atrial cardiopathy, those with atrial cardiopathy were older (54.85 ± 11.86 vs. 43.03 ± 13.78 years, *p =* 0.0003), had a higher prevalence of hypertension (30.00% vs. 11.69%, *p =* 0.0255), and a higher prevalence of diabetes mellitus (10.00% vs. 1.30%, *p =* 0.0146). Following fully adjusted multivariate logistic analysis, atrial cardiopathy (OR = 0.119; *P =* 0.046), BMI (OR = 0.875; *P =* 0.025) and atrial septal aneurysm (OR = 5.465; *P =* 0.028) were identified as independent predictors for residual right-to-left shunt.

**Conclusions:**

The presence of atrial cardiopathy in patients with severe migraine and PFO was inversely associated with residual shunting following PFO closure.

## Introduction

The prevalence of primary headaches in the adult Chinese population is approximately 23.8% [1]. The association between patent foramen ovale (PFO) and migraine-type headaches has been well-established in multiple studies [2, 3]. The study suggests that the incidence of migraine in the PFO population is 5.13 times higher than in the general population, while the incidence of PFO in migraine patients is 2.54 times higher than in the general population. Moreover, the incidence of PFO in patients with unexplained stroke combined with migraine is as high as 79% [4]. The pathogenesis of PFO-induced migraine remains unclear, with two recognized theories: one suggests that vasoactive substances (such as 5-hydroxytryptamine, which are typically metabolized or cleared by the pulmonary circulation) trigger migraine by entering the left heart system and the common carotid artery circulation directly through the PFO. The other theory posits that paradoxical embolism leads to transient cerebral arterial occlusion or hypoperfusion in the regions supplied by the cerebral arteries, resulting in subclinical brain infarctions and localized neurological symptoms, including migraine [5]. An increasing number of studies have shown that percutaneous PFO closure has a palliative effect on migraine. However, in clinical practice, residual shunts may occur in up to 25% of patients following PFO closure, with moderate to severe residual shunts affecting nearly 10% of patients [6, 7]. Karagianni et al. demonstrated that patients with residual shunts faced more than twice the risk of recurrent cerebrovascular events compared to those without residual shunts [8].

Large residual shunts are associated with an increased risk of stroke recurrence, transient ischemic attacks, and headache [9]. In recent years, the concept of atrial cardiopathy has gradually gained attention in research on embolic strokes of undetermined source. Recent studies have suggested that abnormalities in the left atrium, in the absence of atrial fibrillation (AF), may increase the risk of stroke, either as a precursor to AF or as an independent risk factor for atrial thrombosis and subsequent stroke development [10]. Emerging evidence suggests that atrial cardiopathy and pathogenic PFO are two competing etiologic factors in patients with embolic strokes of undetermined source, highlighting that both atrial cardiopathy and PFO serve as underlying potential mechanisms for these strokes. Therefore, a deeper understanding of the relationship between PFO and atrial cardiopathy is essential. Furthermore, the underlying clinical mechanisms of residual shunting remain unclear, and it is crucial to identify the factors associated with residual shunts. In this context, we aimed to explore the relationship between atrial cardiopathy and residual shunt in patients who suffer from migraines and underwent PFO closure. We hypothesized that the presence of atrial cardiopathy is inversely associated with residual shunts.

## Methods

### Study population

A retrospective analysis comparing postoperative residual shunts in PFO patients with and without atrial cardiopathy was conducted. The study cohort consisted of 174 patients with severe migraine and confirmed right-to-left shunt (RLS) (grades II-IV) who voluntarily opted for PFO closure between April 1, 2021, and December 31, 2022. Enrolled patients did not improve with medication for migraine and had few traditional vascular risk factors (e.g., hypertension, diabetes, hyperlipidemia, or smoking). Moreover, patients underwent cranial CT or magnetic resonance examination and were consulted by an experienced neurologist at our hospital to exclude other causes of migraine [11]. Patients were categorized into two groups: PFO with or without atrial cardiopathy. Ethical considerations were followed, with the study adhering to the Declaration of Helsinki and obtaining approval from the Ethics Committee of the First Affiliated Hospital of Dalian Medical University (PJ-KS-KY-2024-710). Informed consent was obtained from all patients in this study.

The presence of PFO was evaluated using contrast transthoracic echocardiography, both with and without the Valsalva maneuver, and, if necessary, via transesophageal echocardiography. The headache impact test (HIT-6) score is a simple self-report assessment system encompassing 6 content categories (pain, social functioning, role functioning, vitality, cognitive functioning, and psychological distress), widely used in headache impact surveys. Patients completed a standardized migraine burden HIT-6 questionnaire at baseline and follow-up. The questionnaire was designed according to the International Headache Society guidelines for migraine, with or without aura [12].

### Definition of atrial cardiopathy

Atrial cardiopathy is a term used to describe structural and pathophysiological changes in the atria, and various electrocardiograms, echocardiograms, and serum biomarkers are associated with the definition of atrial cardiopathy [13, 14]. In the present study. Atrial cardiopathy was defined as an increase in left atrial diameter index (> 23 mm/m^2^) [15] or left atrial volume index (> 34 mL/m^2^) [16], or a prolonged PR interval (≥ 200 ms) [17], or presence of supraventricular extrasystoles, as identified in electrocardiograms performed during hospitalization for the index stroke [18].

### PFO closure

Through a transcatheter approach, successful PFO closure was achieved in all patients using the Amplatzer PFO occluder (Abbott Laboratories, Chicago, Illinois). Routine transthoracic echocardiography and 12-lead electrocardiography were performed 24 h post-procedure and continued until discharge. Following the procedure, patients received a daily dose of 100 mg aspirin for 6 months and a daily 75 mg dose of clopidogrel for 1 month.

### Study endpoints and follow-up

The primary endpoint of this study was the incidence of residual shunt post-PFO closure. All patients underwent clinical follow-up, including serial transthoracic echocardiograms at 1 day, 1-month, 3-month, and 6-month post-device implantation, followed by annual assessments thereafter*.* Clinical follow-up information was obtained through regular clinic visits, telephone interviews utilizing a standardized questionnaire to assess the impact of migraine and a comprehensive review of electronic medical records.

Residual left–right shunt was determined by positive bubble appearance on transthoracic echocardiography 6-month post-PFO closure. In patients with suboptimal transthoracic echocardiography imaging, we further performed transesophageal echocardiography to assess the degree of residual shunting. A mild shunt was defined as the presence of 1 to 9 bubbles in the left atrium, a moderate shunt was defined as 10 to 30 bubbles and a major shunt was defined as > 30 bubbles. Residual shunts were assessed both before and 6 months after PFO closure by cardiologists specializing in echocardiography, who were blinded to the study.

### Statistical analysis

Statistical analysis was conducted using SPSS Statistical Software, Version 26.0. Categorical variables were presented as percentages. Continuous variables with a normal distribution were expressed as mean ± SD, while those with a non-normal distribution were presented as median and interquartile range. Logistic regression analysis was employed to identify independent factors for evaluating the association between atrial cardiopathy and residual shunt. The covariates included in the multivariate logistic analysis comprised independent predictors that demonstrated statistical significance in the univariate logistic analysis. Estimates were expressed as odds ratio (OR) and 95% confidence intervals (CIs). The level of statistical significance was set at 5%.

## Results

### Demographic and clinical characteristics

A total of 174 individuals with migraines who underwent PFO closure (PFO with atrial cardiopathy group, *n =* 20; PFO without atrial cardiopathy group, *n =* 154) were included. Atrial septal aneurysm was detected in 11 (6.32%) patients.

In comparison with patients without atrial cardiopathy, those with atrial cardiopathy exhibited older age (54.85 ± 11.86 vs. 43.03 ± 13.78 years, *p =* 0.0003), and a higher prevalence of hypertension (30.00% vs. 11.69%, *p =* 0.0255), and diabetes mellitus (10.00% vs. 1.30%, *p =* 0.0146), which is consistent with the findings of Zhang et al. [19] (Table 1). Especially, the BMI (26.46 ± 2.945 vs. 23.32 ± 3.063 kg/m^2^, *p* < 0.001), PR interval (166.4 ± 23.71 vs. 154.0 ± 18.46 ms, *p =* 0.0071), LAVI (35.57 ± 3.271 vs. 25.08 ± 4.586 mL/m^2^, p < 0.001), left atrial diameter (37.18 ± 1.741 vs. 31.98 ± 2.895, p < 0.001) and E/e′ (7.544 ± 1.507 vs. 6.150 ± 1.616, *p =* 0.0013) was higher in the PFO with atrial cardiopathy group compared to the PFO without atrial cardiopathy group. Other clinical indicators were not statistically significant between the two groups (P > 0.05).

**Table 1:** Demographic and clinical characteristics

Characteristics	All patients (*n =* 174)	PFO with atrial cardiopathy (*n =* 20)	PFO without atrial cardiopathy (*n =* 154)	*P* value
Age (years), mean (SD)	44.39 ± 14.06	54.85 ± 11.86	43.03 ± 13.78	0.0003
Female	137 (78.73%)	14 (70.00%)	123 (79.87%)	0.3102
BMI	23.68 ± 3.203	26.46 ± 2.945	23.32 ± 3.063	< 0.0001
PR interval	155.4 ± 19.46	166.4 ± 23.71	154.0 ± 18.46	0.0071
Baseline HIT-6	59.07 ± 8.367	56.00 ± 11.14	59.41 ± 7.975	0.1222
Follow-up HIT-6	39.71 ± 7.067	36.30 ± 1.342	40.15 ± 7.385	0.0215
History, No. (%)				
History of smoking	25 (14.36%)	5 (25.00%)	20 (12.99%)	0.1496
History of alcohol consumption	12 (6.89%)	2 (10.00%)	10 (6.49%)	0.5604
Hypertension	24 (13.79%)	6 (30.00%)	18 (11.69%)	0.0255
Diabetes mellitus	4 (2.29%)	2 (10.00%)	2 (1.30%)	0.0146
Atrial septal aneurysm	11 (6.32%)	1 (5.00%)	10 (6.49%)	0.7962
Laboratory values, median (IQR)				
Platelet count, × 10^9^/L	233.4 ± 58.94	246.7 ± 85.30	231.6 ± 54.76	0.2847
PDW, fL	12.27 ± 2.064	12.37 ± 2.206	12.25 ± 2.052	0.8116
MPV, fL	10.40 ± 0.8533	10.19 ± 0.6719	10.43 ± 0.8722	0.2380
PCT	0.2409 ± 0.05421	0.2486 ± 0.07574	0.2399 ± 0.05101	0.5029
P-LCR, %	28.09 ± 6.940	26.57 ± 5.458	28.28 ± 7.101	0.3002
APTT, s	25.66 ± 3.732	24.15 ± 3.764	25.86 ± 3.694	0.0530
PT, s	10.75 ± 0.6232	10.67 ± 0.4769	10.76 ± 0.6403	0.5491
Fibrinogen, g/L	2.528 ± 0.5150	2.807 ± 0.5919	2.491 ± 0.4948	0.0096
Thrombin time, s	16.69 ± 0.9217	16.42 ± 0.5961	16.72 ± 0.9517	0.1649
FDP, mg/L	1.372 ± 1.237	1.892 ± 1.456	1.305 ± 1.194	0.0457
INR	0.9463 ± 0.06018	0.9365 ± 0.04614	0.9476 ± 0.06178	0.4395
Echocardiography findings, median (IQR)				
LAVI	26.16 ± 5.484	35.57 ± 3.271	25.08 ± 4.586	< 0.0001
Left atrial diameter, mm	32.51 ± 3.209	37.18 ± 1.741	31.98 ± 2.895	< 0.0001
E/e′	6.297 ± 1.656	7.544 ± 1.507	6.150 ± 1.616	0.0013
Device size, *n* (%)				
18 mm	1 (0.57%)	0 (0.00%)	1 (0.65%)	
25 mm	167 (95.98%)	19 (95.00%)	148 (96.10%)	
35 mm	6 (3.45%)	1 (5.00%)	5 (3.25%)	

BMI, body mass index; PDW, platelet distribution width; MPV, mean platelet volume; PCT, plateletcrit; P-LCR, platelet-large cell ratio; APTT, activated partial thromboplastin time; PT, prothrombin time; FDP, fibrin degradation products; INR, international normalized ratio; LAVI, left atrial diameter index

### Follow-up efficacy evaluation of transcatheter PFO closure for migraine

**Fig. 1:**
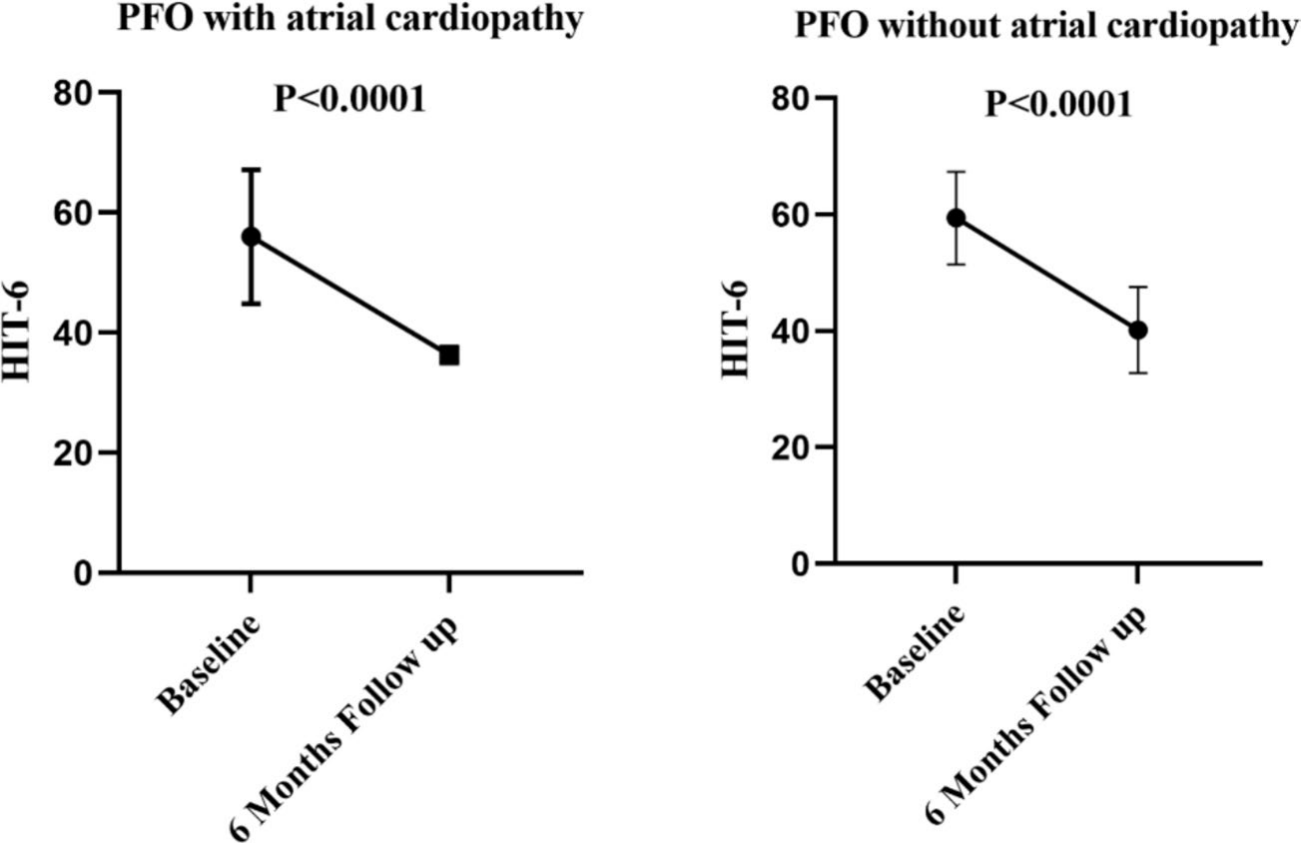
HIT-6 scores in PFO patients with or without atrial cardiopathy. PFO, patent foramen ovale; HIT-6, headache impact test-6

At 6 months following PFO closure, the average HIT-6 scores for patients with atrial cardiopathy (*n =* 20) and those without atrial cardiopathy (*n =* 154) were 36.30 ± 1.342 and 40.15 ± 7.385, respectively, compared to the average HIT-6 scores at baseline of 56.00 ± 11.14 and 59.41 ± 7.975, respectively (Fig. 1).

**Fig. 2:**
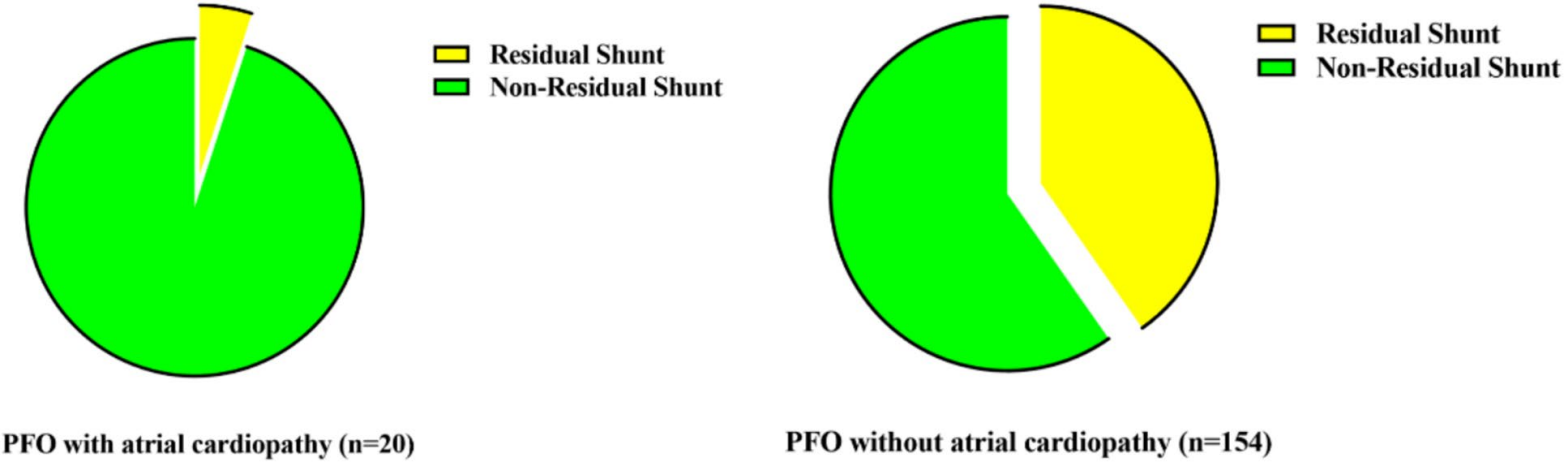
Residual Shunt in PFO patients with or without atrial cardiopathy. PFO, patent foramen ovale

In addition, at the 6-month follow-up, these patients underwent reexamination using contrast transthoracic echocardiography, revealing that 63 (36.20%) patients had residual shunts. Herein, one subject (mild residual shunt) in the PFO with atrial cardiopathy group and 62 in the PFO without atrial cardiopathy group experienced residual right-to-left shunt. In the PFO without atrial cardiopathy group, the residual shunt was mild in 40 patients, moderate in 19 patients, major in 3 patients (Fig. 2).

### Independent predictors for residual right-to-left shunt events

Univariate logistic analysis revealed that atrial cardiopathy (OR, 0.078; 95% CI 0.010–0.599; *P =* 0.014), age (OR, 0.972; 95% CI 0.950–0.995; *P =* 0.017), atrial septal aneurysm (OR, 5.236; 95% CI 1.336–20.526; *P =* 0.018), BMI (OR, 0.839; 95% CI 0.753–0.935; *P =* 0.001), fibrinogen (OR, 0.402; 95% CI 0.203–0.798; *P =* 0.009) and thrombin time (OR, 1.546; 95% CI 1.087–2.198; *P =* 0.015) were independent predictors for residual right-to-left shunt. Following a fully adjusted multivariate logistic analysis, it was found that atrial cardiopathy (OR, 0.119; 95% CI 0.015–0.960; *P =* 0.046), BMI (OR, 0.875; 95% CI 0.779–0.983; *P =* 0.025) and atrial septal aneurysm (OR, 5.465; 95% CI 1.198–24.930; *P =* 0.028) remained independent predictors for residual right-to-left shunt (Table 2).

**Table 2:** Logistic regression analysis to identify factors predicting residual shunt

	Univariate analysis			Multivariate analysis		
	OR	95% CI	*P*	OR	95% CI	*P*
Atrial Cardiopathy	0.078	0.010–0.599	0.014	0.119	0.015–0.960	0.046
Female	1.704	0.763–3.802	0.193	1.394	0.569–3.419	0.467
Age	0.972	0.950–0.995	0.017	0.981	0.955–1.007	0.143
Atrial Septal Aneurysm	5.236	1.336–20.526	0.018	5.465	1.198–24.930	0.028
BMI	0.839	0.753–0.935	0.001	0.875	0.779–0.983	0.025
Fibrinogen	0.402	0.203–0.798	0.009	0.666	0.261–1.701	0.395
Thrombin time	1.546	1.087–2.198	0.015	1.399	0.974–2.008	0.069

BMI, body mass index

## Discussion

This study contributes additional insights and a novel perspective to understanding the association between atrial cardiopathy and residual shunt after PFO closure. Our findings indicate an inverse relationship between the presence of atrial cardiopathy in PFO patients and the occurrence of residual shunting after PFO closure. This implies the existence of alternative mechanisms that may interact with atrial cardiopathy, influencing the persistence of residual shunting following PFO closure. Notably, our findings revealed a significant reduction in HIT-6 scores 6 months after PFO closure, regardless of the presence or absence of comorbid atrial cardiopathy.

Recent studies suggest that abnormalities in the left atrium may increase the risk of stroke in the absence of AF, serving as both a precursor to AF (atrial cardiopathy) and an independent risk factor for atrial thrombosis and subsequent stroke development [13]. Atrial cardiopathy is often associated with comorbidities, such as diabetes mellitus, hypertension, and coronary artery disease. Furthermore, there is an increased likelihood of systemic and local inflammation in these patients, which predisposes them to recurrent major adverse cardiovascular and cerebrovascular events, including stroke [20–22]. Determining the relationship between atrial cardiopathy and residual shunting has proven challenging, and further research is warranted to establish a consensus definition for atrial cardiopathy, which currently lacks uniformity [23]. Several parameters with varying thresholds have been used to define atrial cardiopathy, including echocardiography, electrocardiography, cardiac magnetic resonance imaging, and serum biomarkers; however, it remains unclear which are most optimal [23]. The presence of atrial cardiopathy is inversely associated with the presence of likely pathogenic PFO in patients with embolic stroke of undetermined source [18]. In this study, atrial cardiomyopathy is inversely associated with residual shunts after PFO closure, as evidenced by multivariate logistic analysis.

It has been found that residual shunts are present in 10–20% of patients 1 year after PFO closure. Xing et al. found that migraine relief was more pronounced in patients without residual shunts compared with those with residual shunts, suggesting that residual shunts have an impact on the degree of migraine relief. Follow-up to 6 months and 1 year found that the number of patients with residual shunts decreased at 1 year compared to 6 months, suggesting that endothelialization of the PFO closure cannot be fully formed in 6 months, and that the follow-up time can be extended for patients with postoperative residual shunts. Indeed, in this study, we found a higher amount of residual shunt in patients at 6 months of follow-up, and in future studies we will follow such patients for a longer period of time. Rovera et al. found that there was no statistically significant difference between the residual shunt group compared to the patients in the no residual shunt group in terms of atrial septal aneurysm preoperative size of right-to-left shunt, size of the blocker device, and type of blocker device [7]. This study found that atrial septal aneurysm and lower BMI were independent predictors of residual shunt. This is consistent with the findings of Greutmann et al., who found that the presence of an atrial septal aneurysm significantly increased the residual shunt rate at the 6-month follow-up [24]. In PFO patients with low BMI, the larger surface area of the occluder device may delay endothelialization [24]. The presence of traditional risk factors may contribute to a persistent residual shunt over time, potentially by hindering epithelialization after PFO closure, thereby increasing the patient's risk of paradoxical embolism [25]. Regarding the possible reasons for the beneficial effect of atrial cardiopathy on residual shunting, we consider that atrial cells (both cardiomyocytes and noncardiomyocyte components, such as fibroblasts, endothelial cells, and nerve fibers) respond rapidly and extensively to pathologic stimuli and are susceptible to a range of genetic factors. The effect of atrial fibroblast overproliferation on the endothelialization process after PFO closure could be a focus for future studies on factors influencing residual shunting.

### Limitations

First, the retrospective design and patient self-selection for PFO closure may introduce selection bias. Second, our definition of atrial cardiopathy was based on clinically relevant electrocardiogram and echocardiographic markers of left atrial enlargement and dysfunction; however, several conditions and markers for atrial disease associated with atrial cardiopathy (such as p-wave terminal force in lead V1, brain natriuretic peptide) were not included in the analysis. Third, endothelialization of the PFO occluder may not yet be complete, as this study did not provide supporting information on the anatomical characteristics of the PFO (size, associated defects, tunnel length, etc.), nor did it include 1-year follow-up data. In future studies, these patients should be evaluated with long-term follow-up.

## Conclusion

The present study revealed an inverse association between the presence of atrial cardiopathy in migraineurs with PFO and the occurrence of residual shunting following PFO closure. Collectively, our findings contribute valuable insights that may guide the development of targeted diagnostic tests and treatment strategies for managing postoperative residual shunts after PFO closure in this particular population.

## Data Availability

No datasets were generated or analysed during the current study.
